# Subunit composition of CP43-less photosystem II complexes of *Synechocystis* sp. PCC 6803: implications for the assembly and repair of photosystem II

**DOI:** 10.1098/rstb.2012.0066

**Published:** 2012-12-19

**Authors:** M. Boehm, J. Yu, V. Reisinger, M. Beckova, L. A. Eichacker, E. Schlodder, J. Komenda, P. J. Nixon

**Affiliations:** 1 Division of Molecular Biosciences, Imperial College London, South Kensington Campus, London SW7 2AZ, UK; 2 Center for Organelle Research (CORE), University of Stavanger, Kristine Bonnevis vei 22, 4036 Stavanger, Norway; 3 Department of Phototrophic Microorganisms, Institute of Microbiology, Academy of Sciences, 37981 Třeboň, Czech Republic; 4 Faculty of Science, University of South Bohemia, Branisovska 31, Ceske Budejovice, Czech Republic; 5 Max-Volmer Laboratory for Biophysical Chemistry, Technical University Berlin, 10623 Berlin, Germany

**Keywords:** RC47, *Synechocystis*, low-molecular-mass subunit, accessory factor, Psb28, ScpC

## Abstract

Photosystem II (PSII) mutants are useful experimental tools to trap potential intermediates involved in the assembly of the oxygen-evolving PSII complex. Here, we focus on the subunit composition of the RC47 assembly complex that accumulates in a *psbC* null mutant of the cyanobacterium *Synechocystis* sp. PCC 6803 unable to make the CP43 apopolypeptide. By using native gel electrophoresis, we showed that RC47 is heterogeneous and mainly found as a monomer of 220 kDa. RC47 complexes co-purify with small Cab-like proteins (ScpC and/or ScpD) and with Psb28 and its homologue Psb28-2. Analysis of isolated His-tagged RC47 indicated the presence of D1, D2, the CP47 apopolypeptide, plus nine of the 13 low-molecular-mass (LMM) subunits found in the PSII holoenzyme, including PsbL, PsbM and PsbT, which lie at the interface between the two momomers in the dimeric holoenzyme. Not detected were the LMM subunits (PsbK, PsbZ, Psb30 and PsbJ) located in the vicinity of CP43 in the holoenzyme. The photochemical activity of isolated RC47-His complexes, including the rate of reduction of P680^+^, was similar to that of PSII complexes lacking the Mn_4_CaO_5_ cluster. The implications of our results for the assembly and repair of PSII *in vivo* are discussed.

## 1. Introduction

The photosystem II (PSII) complex is the light-driven water : plastoquinone oxidoreductase of oxygenic photosynthesis, located in the thylakoid membranes of cyanobacteria and chloroplasts [1]. The recent X-ray crystal structures of dimeric PSII complexes isolated from thermophilic cyanobacteria, at resolutions of 3.5 Å [2], 3.0 Å [3], 2.9 Å [4] and 1.9 Å [5], have provided remarkable insights into the organization of the proteins and cofactors within the oxygen-evolving holoenzyme. However, the molecular details of PSII assembly remain largely unknown. Recent work has led to the proposal that PSII is assembled in a step-wise manner from smaller sub-complexes or modules consisting of a large chlorophyll (Chl)-binding protein (D1, D2, CP43 or CP47) and one or more low-molecular-mass (LMM) subunits plus bound pigment [6]. According to this model, a PSII reaction centre (RC) complex is assembled from PsbI/precursor D1 [7] and cytochrome (cyt) *b*-559/D2 [8] sub-complexes. Attachment of the CP47 sub-complex [9] results in formation of an assembly intermediate called the RC47 assembly complex [8], which then binds the CP43 sub-complex [9] to form the monomeric PSII core complex. At this stage, the oxygen-evolving Mn_4_CaO_5_ cluster is able to assemble in a light-driven process, the lumenal extrinsic proteins (PsbO, PsbU and PsbV) are attached and PSII can dimerize. A number of auxiliary factors have also been identified that are important for assisting/regulating assembly [10–12].

PSII is also a weak link in photosynthesis and is vulnerable to irreversible damage by visible light *in vivo*, leading to so-called chronic photoinhibition [13]. Damaged PSII can, however, be repaired through the operation of a ‘PSII repair cycle’ in which the damaged protein subunit, mainly the D1 subunit, is selectively replaced by a newly synthesized subunit and PSII reactivated [12]. Current models suggest that following damage, dimeric PSII partially disassembles and damaged D1 is removed from a monomeric PSII sub-complex lacking the lumenal extrinsic proteins and the CP43 complex [14–16]. Once damaged D1 has been replaced, active dimeric PSII complexes are reassembled following reattachment of CP43 and the extrinsic subunits.

A PSII core complex lacking CP43, often referred to as the RC47 complex in cyanobacteria [8], or sometimes the CP43-less core monomer in chloroplasts [17], can be detected in mildly solubilized thylakoid membrane extracts by blue native–polyacrylamide gel electrophoresis (BN–PAGE). Increased amounts of RC47 are found in mutants with impaired binding of CP43, such as *Δ*PsbI [18] and *Δ*PsbK [10], and in strains with impaired PSII repair [19,20]. The ‘RC47 complex’ detected in thylakoid membranes by BN–PAGE is, however, potentially heterogeneous, as CP43-less PSII core complexes are formed in both the PSII repair cycle and during de novo assembly.

A major impediment to isolation and detailed characterization of the RC47 assembly complex is its low abundance in the thylakoid membrane and potential contamination by RC47 complexes generated from disassembly of PSII during repair or during sample preparation. It is, however, likely that the RC47 assembly complex is very similar in composition to the non-oxygen-evolving PSII sub-complex that accumulates in mutant strains unable to synthesize the CP43 subunit [21]. Despite only being expressed at 10 per cent of wild-type (WT) levels, Rögner *et al.* [22] were able to purify CP43-less PSII complexes from a *psbC* deletion mutant of *Synechocystis* 6803, using first an anion exchange and then a hydroxyapatite chromatography step. They showed that the isolated complex was monomeric and was inactive in oxygen evolution, but was still able to catalyse light-driven electron transfer from tyrosine Y_z_ to the primary quinone electron acceptor, Q_A_ [22].

Here, we have extended the pioneering studies of Rögner *et al.* [22] to include a detailed assessment of the oligomerization state and subunit composition of the RC47 assembly complex *in vivo.* In addition, we have used a His-tagging approach to isolate the RC47 assembly complex to permit analysis of its subunit composition and photochemical activity. The implications of our results for the assembly and repair of PSII *in vivo* are discussed in light of the recent advances in our understanding of the structure of the cyanobacterial PSII holoenzyme.

## 2. Material and methods

### (a) Cyanobacterial strains and growth conditions

The glucose-tolerant strain of *Synechocystis* sp. PCC 6803 [23] and the previously constructed *Δ*CP43 [21] and His-tagged CP47 (PSII-His) strains [9] were used in this work. Strains were grown in liquid BG-11 mineral medium and maintained on solid BG-11 plates containing 1.5 per cent (w/v) agar, both containing 5 mM *N*-tris (hydroxymethyl)methyl-2-aminoethanesulfonic acid–KOH, pH 8.2, at a light intensity of 40 or 5 µE m^−2^ s^−1^ of white fluorescent light, respectively and at 29°C. The medium was supplemented with 5 mM glucose and where applicable, kanamycin (50 µg ml^−1^) or erythromycin (10 µg ml^−1^) was added.

### (b) Construction of mutants

To generate a *Synechocystis* sp. PCC 6803 mutant strain that contained His-tagged PSII lacking CP43 (strain *Δ*CP43/CP47-His), the CP47 protein was His-tagged, and the CP43 protein was inactivated by partial deletion of the *psbC* gene and replacement by a kanamycin-resistance cassette. To His-tag the CP47 protein, the gentamycin-resistance cassette of the pCP47His-tagGm^R^ plasmid [24] was removed by *Bam*HI digestion and, after blunting the ends, an erythromycin-resistance cassette was introduced to generate pCP47His-Ery^R^. This plasmid was transformed into the *Synechocystis* sp. PCC 6803 glucose-tolerant strain to generate the PSII-His mutant. To inactivate the CP43 protein, the *psbC* gene was amplified by PCR with the following primers: CP43 + 1000-Fw, 5′-ATATTTTCCCCTTCTTCGTAGGGGTGC-3′ and CP43 + 1000-Rev, 5′-CTGCCATTAAAGAATTGGCTAAAGAAGCAGGTC-3′. After ligation into the pGEMTeasy vector (Promega, UK), a kanamycin-resistance cassette was introduced between the *Hin*dIII and *Sma*I sites located between 767 bp and 1228 bp, respectively, downstream of the start codon annotated in CyanoBase. This plasmid was transformed into the CP47His mutant to yield the CP47-His/*Δ*PsbC strain. The genotypes of the mutants were verified by PCR analysis, using gene-specific primers.

Plasmid pPsbAIpetJ-FLAG was used for construction of the strain expressing FLAG-tagged Psb28-2 under the control of the copper-regulated *petJ* promoter at the *psbAI* locus. It was constructed as follows: a 600-bp *Xba*I-*Cfr*9I fragment upstream and a 600-bp *Bgl*II–*Xma*jI fragment downstream of the *psbAI* gene were amplified by PCR and cloned into pETBlue-2 plasmid (Novagen). The *petJ* promoter from *Synechocystis* sp. PCC 6803 (positions 846 614–846 331 according to CyanoBase) and 3xFLAG sequence (Sigma) were amplified by PCR, ligated and again amplified by PCR for cloning between the *psbAI* fragments. Finally, the kanamycin-resistance gene (*aphX*) from pUC4K, amplified as a *Bam*HI-*Bgl*II fragment, was cloned into the *Bgl*II site upstream of the second *psbAI* fragment leaving a single *Bgl*II site for cloning of *psb28-2*.

To clone FLAG-tagged Psb28-2 into the integration plasmid, the *psb28-2* (*slr1739*) gene was amplified by PCR using the following primers: *Not*I + *slr1739-*Fw, 5′-CCGGTGGCGGCCGCAATGACCCTCACTCCC-3′ and *slr1739* + *Bgl*II-Rev, 5′-AACTTTAGATCTCTAACGATCTTGGTAG-3′. After *Not*I and *Bgl*II digestion and ligation into the plasmid, the construct was transferred into the *Δ*Psb28-2 deletion mutant. This mutant was previously constructed by replacement of the *psb28-2* gene by a chloramphenicol-resistance cassette. Segregation in the *psbAI* locus was confirmed by PCR analysis, using gene-specific primers. To induce expression of the FLAG-tagged Psb28-2 protein, the strain was cultivated in BG-11 medium with 5 mM glucose lacking CuSO_4_.

### (c) Isolation of protein complexes

The RC47-His protein complex was purified by Ni^2+^-affinity chromatography as described for the CP43-His and CP47-His proteins [9]. However, the fractions eluted with 50 and 100 mM imidazole were concentrated using 100 kDa molecular weight cut-off (MWCO) protein concentrators (Sartorius, UK). As a second purification step, the concentrated affinity-purified sample was diluted 10 times with KPN buffer (40 mM K-phosphate, pH 8.0, 100 mM NaCl) containing 0.04 per cent (w/v) *n*-dodecyl-β-d-maltoside (β-DM) and loaded onto a column packed with Toyopearl 650S DEAE anion-exchange chromatography resin (Anachem, UK). Chromatography was performed at a flow rate of 0.5 ml min^−1^ and with KPN buffer containing 0.04 per cent (w/v) β-DM as the running buffer. Initially, for the first 10 min, the running buffer also contained 5 mM MgSO_4_ and over the next 50 min, the concentration of MgSO_4_ was raised linearly to 200 mM. The run was monitored at 280 nm using a Jasco MD-2015 plus diode array detector (Jasco, UK) and 0.5-ml fractions were collected by a Frac-920 fraction collector (GE Healthcare, UK). Selected fractions were pooled, supplemented with 10 per cent (v/v) glycerol and concentrated using 100-kDa MWCO protein concentrators (Sartorius, UK).

His-tagged oxygen-evolving and non-oxygen-evolving PSII complexes were isolated from the PSII-His strain, using the methods described by Service *et al.* [25] and Boehm *et al.* [9], respectively.

For isolation of Flag-tagged Psb28-2, membranes were solubilized in KPN buffer containing 1 per cent β-DM, and the supernatant was loaded onto a column containing 300 µl of anti-FLAG M2 affinity gel (Sigma, USA), pre-equilibrated with KPN buffer containing 0.04 per cent β-DM (KPN–DDM). To remove any loosely bound contaminants, the column was first washed with 5 ml of KPN–DDM and then the FLAG-Psb28-2 was eluted by a 30 min incubation of resin in 200 µl of KPN-DDM containing 20 per cent glycerol and 150 µl ml^−1^ 3xFLAG peptide (Sigma, USA). Resin was removed by centrifugation at 500*g* for 5 min. His-tagged Psb28 was isolated as described in Dobakova *et al*. [26].

### (d) Protein analysis

The Chl *a* content of samples was determined by extraction into methanol and absorption measurements at 666 and 750 nm [16]. Protein samples were analysed by BN– and SDS–PAGE, according to Boehm *et al.* [27]. Unless stated otherwise, 6–12% (w/v) polyacrylamide (PAA) BN–PAGE and 18% (w/v) PAA SDS–PAGE gels containing 6 M urea were used. Alternatively, a combination of 4–14% (w/v) PAA clear native (CN) and 12–20% (w/v) PAA SDS–PAGE containing 7 M urea was used for analysis of membrane protein complexes, as described in Komenda *et al*. [10]. The resulting gels were stained with either Coomassie blue or silver or Sypro Orange or electro-blotted onto PVDF membrane using the iBlot system (Invitrogen, UK), according to the manufacturer's instructions. Immunoblotting analyses were conducted using specific primary antibodies and a horseradish peroxidase-conjugated secondary antibody (GE Healthcare). Signals were visualized using a chemiluminescent kit (SuperSignal West Pico, Pierce, USA). Primary antibodies used in this study were: (i) αD1, αD2, αCP43 and αCP47 [9]; (ii) αHis-tag (Invitrogen); (iii) αPsbH (raised against the whole PsbH protein from *Synechocystis* 6803); (iv) αPsb28 [26]; (v) αPsb28-2 (raised against peptide 40–53 of the Psb28-2 protein from *Synechocystis* 6803); (vi) αPsb28 (raised against Psb28 of *Thermosynechococcus elongatus*); and (vii) αScpC/D [28]. Cells were pulse-labelled for 20 min at 29°C and at a light intensity of 500 µE m^−2^ s^−1^ as described in Komenda *et al*. [10].

### (e) Low-temperature absorption spectroscopy

Spectra were recorded at a resolution of 0.5 or 1 nm in a Cary-1E-UV/vis spectrophotometer (Varian) equipped with a liquid nitrogen bath cryostat (DN 1704 from Oxford). The purified protein complexes were diluted to an optical density (OD) of approximately 1 at the maximum in the Q_Y_-region in buffer (10 mM MES pH 6.5, 10 mM CaCl_2_, 10 mM MgCl_2_, 0.02% β-DM and 60–65% glycerol).

### (f) Detection of cyt *b*-559

Isolated RC47-His complexes containing 10 μg of Chl *a* were diluted into 500 μl of KPN buffer containing 1 μM potassium ferricyanide, and a baseline was run for the sample between 520 and 600 nm (UV-1601, Shimadzu, UK). After the addition of a few grains of sodium dithionite to the sample, the reduced-minus-oxidized absorbance spectrum was recorded.

### (g) Transient absorbance spectroscopy

Laser-flash-induced absorbance changes (*Δ*A) at 830 nm owing to the formation of the Chl radical cation P680^+^ were measured as described previously [29], using a continuous wave laser diode (Hitachi HL 8318G) as the measuring light source. The samples were excited by non-saturating laser flashes at 532 nm with a pulse duration of 3 ns (Nd-YAG laser YG411 from Quantel). For measurements in the nanosecond range, the detection system (photodiode FND-100 from EG and G; amplifier IV86 from HMI; transient recorder Tektronix TDS 540B, sampling rate 2 GS s^−1^) had an electrical bandwidth of 500 Hz–200 MHz. In the micro- and millisecond range, a HVA-10M-60-F amplifier from FEMTO was used. The decay kinetics were analysed by a sum of exponential decay components minimizing the sum of squares of the weighted residuals.

### (h) Mass spectrometry

Mass spectrometry of LMM proteins was performed as described by Granvogl *et al.* [30]. For offline electrospray ionization mass spectrometry (ESI-MS), 20 µl of anion-exchange-purified RC47-His protein complex was precipitated in 80 per cent (v/v) acetone at −20°C overnight. After centrifugation for 20 min at 13 000*g* in a microfuge, the supernatant was discarded and the air-dried pellet dissolved in a solution containing 70 per cent (v/v) acetone, 10 per cent (v/v) 2-propanol and 1 per cent (v/v) formic acid. The sample was then directly applied to a nano spray emitter and mass spectra were obtained using a Waters Q-TOF premier equipped with a nanoESI source. For scanning the LMM proteins, MS spectra at the mass range of 800–2500 *m*/*z* were acquired, and data were recorded at a capillary voltage of 0.8 kV and a cone voltage of 37 V. After MS data collection at a rate of 1 s per scan, 30 scans were averaged. The acquisition of fragment ion spectra was performed at a collision energy between 26 and 40 eV, and data were analysed by the MassLynx/BioLynx v. 4.1 software. Sequence tags obtained from the fragment spectra were used for similarity search (www.ebi.ac.uk/Tools/fasta33), and the sample was analysed in several repetitions.

## 3. Results

### (a) Characterization of the RC47 assembly complex in a *Synechocystis* strain lacking CP43

**Figure 1.**
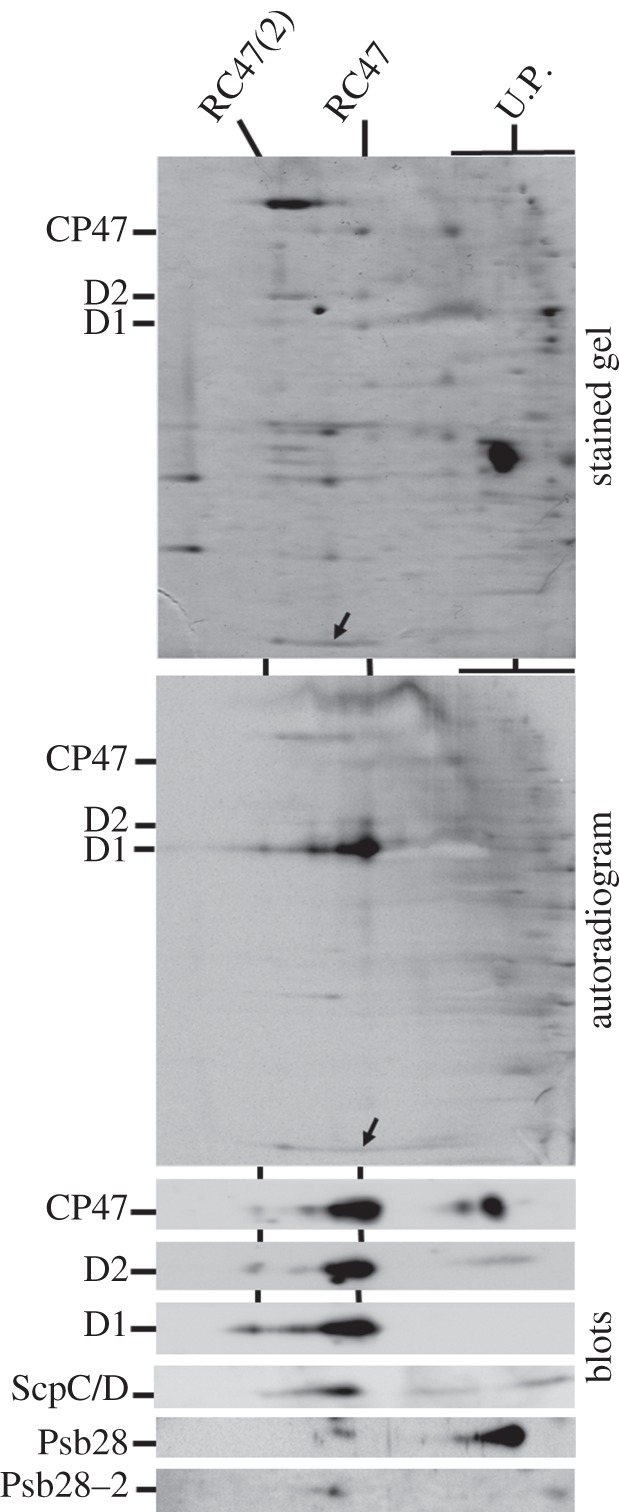
Two-dimensional analysis of thylakoid membranes isolated from the radioactively labelled *Δ*CP43 *Synechocystis* strain. Thylakoid membranes corresponding to an amount of 4 µg Chl *a* were separated on a 4–14% (w/v) polyacrylamide (PAA) CN-PAGE linear gradient gel, and another 12–20% (w/v) PAA SDS–PAGE gel was used for the second dimension. The gels were stained with Sypro Orange (stained gel), then blotted onto PVDF membrane and sequentially probed with antibodies against indicated proteins (blots). Dried membrane was then used for autoradiography (autoradiogram). The positions of monomeric RC47 and dimeric RC47 (RC47(2)) are indicated. U.P. are unassembled proteins; arrows indicate location of the ScpC/D proteins on the gel and autoradiogram.

To assess the oligomeric status of the RC47 assembly complex *in vivo*, we carried out a two-dimensional protein gel analysis of solubilized thylakoids isolated from radiolabelled cells of a *psbC* deletion mutant, *Δ*CP43, unable to synthesize CP43 [8,22]. Protein complexes were resolved by clear-native gel electrophoresis in the first dimension, then by denaturing gel electrophoresis in the second dimension (figure 1). Immunochemical detection of PSII subunits indicated that the RC47 assembly complex was present mainly as the monomer (of size approx. 220 kDa), but that trace amounts of a larger complex, most probably the dimer, were also present (figure 1; RC47(2)). Smearing of the monomeric RC47 band suggested some heterogeneity in composition. Some unassembled CP47 was also observed in the LMM region. Newly synthesized, radiolabelled D1 protein was exclusively detected in the RC47 complex (figure 1, autoradiogram). The high degree of labelling of D1 in comparison with other PSII proteins is consistent with extremely fast turnover of D1 in the RC47 complex (see also Komenda *et al*. [19]).

**Figure 2.**
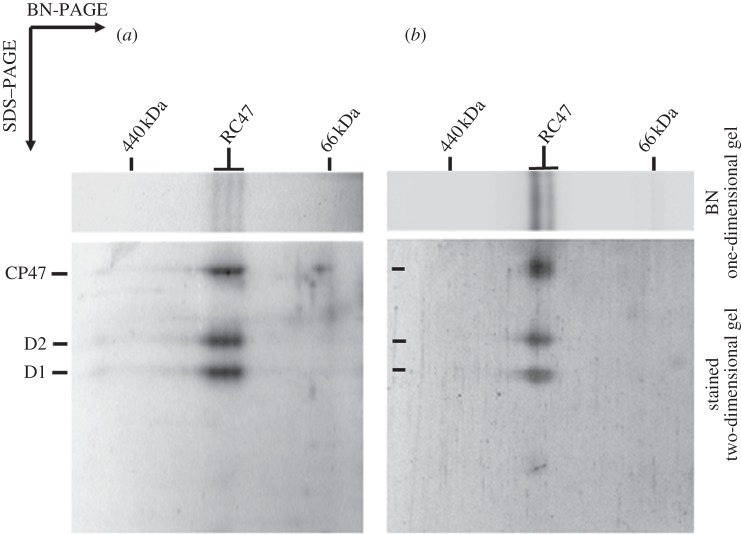
Two-dimensional analysis of RC47 complexes isolated from thylakoid membranes of *Synechocystis* strains expressing (*a*) His-tagged Psb28 or (*b*) FLAG-tagged Psb28-2. Complexes corresponding to an amount of 1 µg Chl *a* were separated on a 4–14% (w/v) polyacrylamide (PAA) CN–PAGE linear gradient gel, and another 12–20% (w/v) PAA SDS–PAGE gel was used for the second dimension. The gels were stained with Sypro Orange. The position of the monomeric RC47 is indicated.

Dobakova *et al.* [26] have previously shown that Psb28 (Sll1398), an accessory protein of PSII not found in the crystallized holoenzyme, is able to associate with the RC47 complex *in vivo*. In line with this conclusion, we were also able to detect Psb28 in the RC47 assembly complex in the *Δ*CP43 mutant, although most Psb28 was found unassembled (figure 1). The genome of *Synechocystis* also contains a gene coding for a second Psb28 homologue termed Psb28-2 (Slr1739). Using specific anti-peptide antibodies, we detected a small amount of Psb28-2 protein co-migrating with the RC47 complex (figure 1, lower panel, blots). To obtain stronger support for a specific association between Psb28-2 and RC47, we conducted pull-down experiments using a strain of WT *Synechocystis* 6803 expressing FLAG-tagged Psb28-2 and were able to affinity-purify RC47 complexes (figure 2). The two RC47 bands separated by native PAGE reflect release of FLAG-tagged Psb28-2 either during electrophoresis or during the isolation procedure (data not shown). Using a similar approach, RC47 complexes could also be affinity-purified using His-tagged Psb28 (figure 2; see also [26]). In this case, the three bands observed by native electrophoresis appear to reflect the presence or absence of His-tagged Psb28 (data not shown).

Upon exposure to high irradiances (500 µE m^−2^ s^−1^), the ScpC/D subunits, which are small Cab-like proteins possibly involved in transient binding of pigments [31], have also been detected in the RC47 complex found in WT samples, both by radioactive labelling and by immunodetection [28,32]. Interestingly, in contrast to WT [32], there was no detectable radioactive labelling of these bands in the *Δ*CP43 mutant (figure 1, upper and middle panels, arrows), indicating that the ScpC/D proteins were either not synthesized under the radiolabelling conditions used or had already preaccumulated in the complex even at a low irradiance of 40 µE m^−2^ s^−1^. The latter alternative was supported by immunoblot analysis using an antibody specific for SpcC/D (figure 1, blots) and further confirmed by detection of ScpC/D in the same cells that were not radioactively labelled and thus not treated for short times at high irradiance (data not shown).

Rögner *et al.* [22] have estimated that the RC47 complex in *Δ*CP43 accumulates to about 10 per cent of the level of PSII core complexes found in WT. However, when we grew the *Δ*CP43 strain at low irradiance (5 µE m^−2^ s^−1^), the cellular content of the RC47 complex strongly decreased, reaching a level hardly detectable by protein staining (see the electronic supplementary material, figure S1). Semi-quantitative immunoblotting, using antibodies against D1, suggested that the amount of RC47 was less than 10 per cent of the level found in the strain grown under our standard growth conditions (40 µE m^−2^ s^−1^; electronic supplementary material, figure S1). These data showed that the accumulation of the RC47 complex in the *Δ*CP43 strain was strongly irradiance-dependent. This effect was specific for the RC47 complex as the amount of PSII core complexes in WT cells cultivated under identical conditions did not change significantly with varying irradiance (see the electronic supplementary material, figure S1). In addition, there was a noticeable decrease in phycobilin content in low-light-grown cells of *Δ*CP43 in comparison with cells grown under standard conditions (see the electronic supplementary material, figure S2).

### (b) Isolation of the RC47-His complex from *Synechocystis* sp. PCC 6803

To perform a more detailed characterization of the RC47 assembly complex, we isolated a His-tagged RC47 complex from a strain of *Synechocystis* 6803 (*Δ*CP43/CP47-His) in which a His_6_-tag was added to the C-terminus of CP47 and the *psbC* gene encoding CP43 was inactivated (see §2). Previous work has shown that a C-terminal His-tag on CP47 does not prevent assembly of a functional PSII complex [33,34]. In agreement with results obtained with the non-tagged strain under standard growth conditions, the level of expression of the RC47-His complex was approximately 10 per cent of the levels of PSII in WT as deduced by two-dimensional BN–PAGE and was mainly present as a monomer of size approximately 220 kDa (data not shown). Despite the relatively low levels of expression, the RC47-His complex could be purified from detergent-solubilized thylakoid membranes by immobilized Ni-affinity chromatography (see the electronic supplementary material, figure S3*a*). Some residual contaminating proteins were removed by a subsequent anion-exchange chromatography step (see the electronic supplementary material, figure S3*b*). BN–PAGE revealed that the final RC47-His complex was largely monodisperse with a size of 220 kDa (see the electronic supplementary material, figure S3*c*).

**Figure 3.**
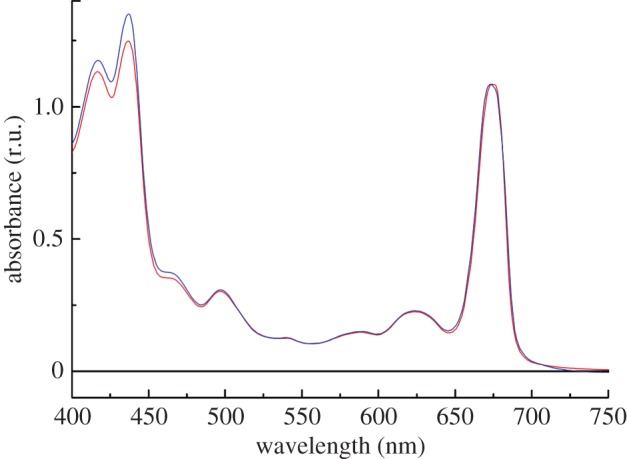
77K absorption spectra of the RC47-His and PSII-His protein complexes isolated from *Synechocystis* sp. PCC 6803. Red, RC47-His; blue, PSII-His.

The 77K absorption spectra of the RC47-His complex and a control non-oxygen-evolving His-tagged PSII complex (PSII-His) are shown in figure 3. The main qualitative difference between the two spectra is a relative reduction in the RC47-His complex of the intensity of the absorption band at 435 nm (Soret transition of Chl *a*) indicative of the presence of less Chl *a* in the complex, as observed previously [22].

### (c) Subunit composition

**Figure 4.**
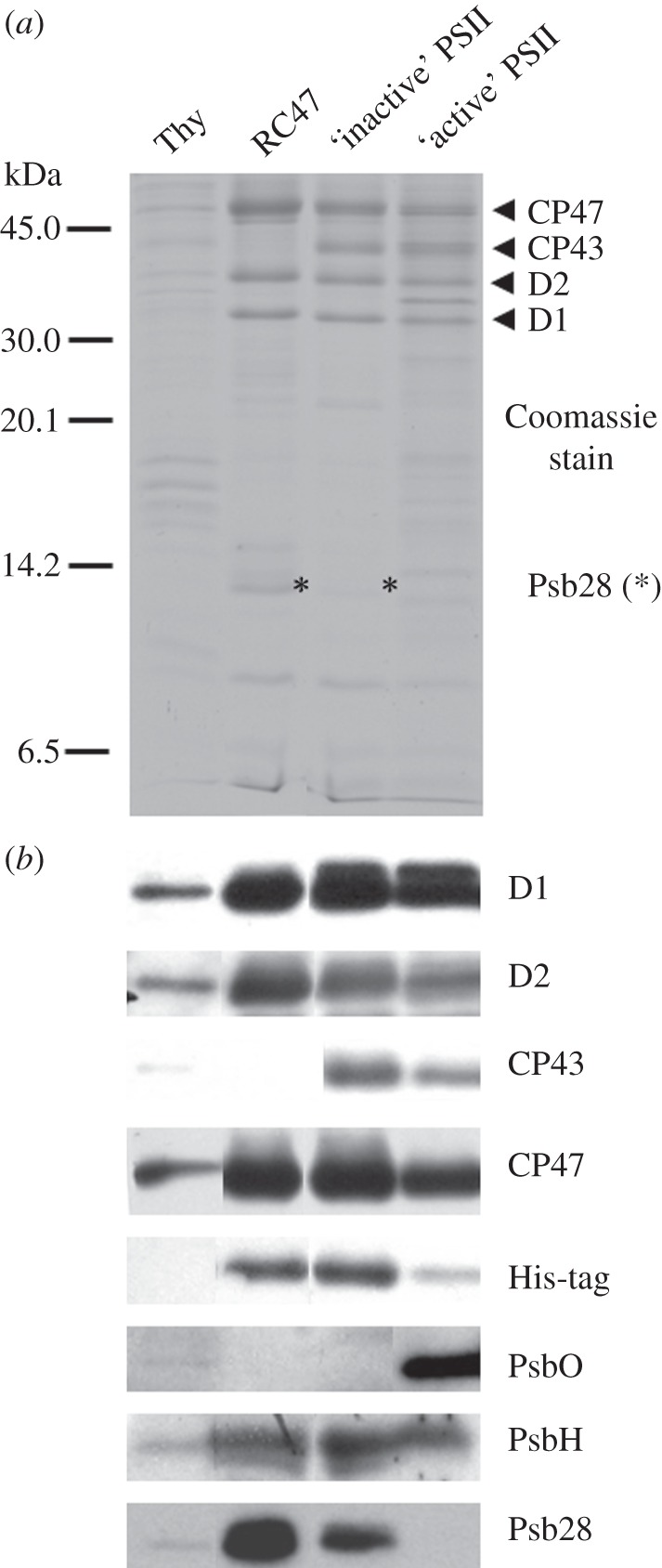
SDS–PAGE and immunoblotting analysis of the RC47-His and PSII-His complexes isolated from *Synechocystis* sp. PCC 6803. (*a*) Coomassie-stained 18% (w/v) PAA SDS–PAGE gel and (*b*) immunoblotting analyses with indicated antibodies. Thylakoid membranes (0.5 µg Chl *a*), the RC47-His protein complex (1 µg Chl *a*) and non-oxygen-evolving (inactive) as well as oxygen-evolving (active) PSII core complexes (1 µg Chl *a* each) were loaded on the gels.

Analysis of the RC47-His complex and control oxygen-evolving (active) and non-oxygen-evolving (inactive) PSII-His complexes by SDS–PAGE is shown in figure 4*a*. The presence of the CP47, D1, D2 and PsbH subunits in the RC47-His complex and the absence of CP43 and PsbO was confirmed by immunoblotting (figure 4*b*). Immunoblotting using antibodies raised to Psb28 from *T. elongatus* indicated that the major 13-kDa Coomassie-blue-stained band present in RC47-His contained Psb28 (figure 4*b*). Mass spectrometry confirmed the presence of both Psb28 and Psb28-2 in this band in line with the results presented in figure 1 (data not shown). The presence of low amounts of Psb28 in the inactive PSII-His complexes might reflect some contamination by RC47-His which co-purifies.

Each cyanobacterial PSII monomer in the crystallized PSII dimeric complex contains 12–13 LMM subunits, depending on the preparation. Two of these subunits, PsbE and PsbF, are the apopolypeptides of cyt *b*-559, which we could detect in the RC47-His complex by reduced-minus-oxidized difference spectroscopy (see the electronic supplementary material, figure S3*d*). Mass spectrometry unambiguously identified a further 7 LMM subunits in the isolated RC47-His complex (see the electronic supplementary material, figure S4 and table 1), including PsbT, PsbM and PsbL, which in the dimeric complex lie at the interface between the two monomeric complexes.

**Table 1.** Detection of low-molecular-mass proteins in the RC47-His complex by mass spectrometry. SU, subunit. Accession number according to the UniProt database (http://www.uniprot.org/).

PSII SU	accession no.	detected signals (charge state)	de novo sequenced protein fragment	sequence coverage (%)
PsbF	P09191	1200.67 [M + 4H]^4+^	VFFVGAIAAMQFIQR	34
PsbH	P14835	1168.22 [M + 6H]^6+^	PVMGVFMALFL	17
PsbI	Q54697	867.41 [M + 5H]^5+^	FISLFIFGFLS	66
		874.99 [M + 5H]^5+^	YVVGLFISLFIFGFLSSD	
		1083.76 [M + 4H]^4+^	TLKIAVYIVVGLFISLFIFGFLS	
		1087.77 [M + 4H]^4+^	YIVVGLFISL	
		1093.25 [M + 4H]^4+^	LKIAVYIVVGLFISLFIFG	
PsbL	Q55354	1118.61 [M + 4H]^4+^	YLGLLLVAVLGILFSSYF	46
		1122.52 [M + 4H]^4+^	YLGLLLVAVLGILF	
		1124.01 [M + 4H]^4+^	LLVAVLGIL	
		1128.03 [M + 4H]^4+^	LLVAVLGIL	
PsbM	P72701	1310.68 [M + 3H]^3+^	VNNLGFIASIL	46
		1316.31 [M + 3H]^3+^	VNNLGFIASILFVLVP	
PsbT	P74787	892.43 [M + 4H]^4+^	LVLTMALAVL	68
		897.88 [M + 4H]^4+^	SVAYILVLTMALAVLFFAI	
		899.41 [M + 5H]^5+^	LVLTMALAVL	
		902.17 [M + 4H]^4+^	ALAVLFFAI	
		1189.42 [M + 3H]^3+^	MESVAYILVLTMALAVLF	
		1194.92 [M + 3H]^3+^	MESVAYILVLTMALA	
PsbX	P72575	1053.87 [M + 4H]^4+^	LGAAIVLIPAT	72
		1059.19 [M + 4H]^4+^	LWSLVLGAAIVLIPATVGLIFISQKDKI	
		1063.27 [M + 4H]^4+^	SLVLGAAIVLIPAT	
		1067.26 [M + 4H]^4+^	LGAAIVLI	
PsbY	P73676	1057.82 [M + 4H]^4+^	RVIVVVSPLLIAATWAAINIG	54

### (d) Electron transfer in the RC47-His complex

**Figure 5.**
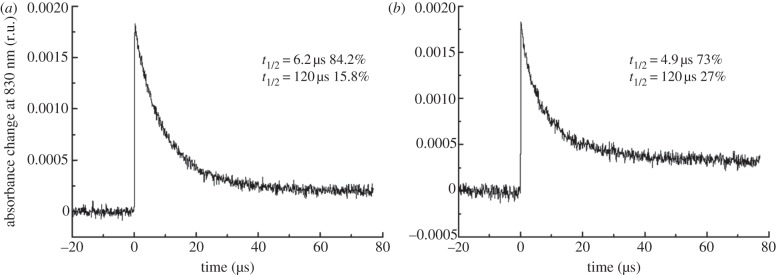
Kinetics of P680^+^ reduction. Laser-flash-induced absorbance changes (*Δ*A) at 830 nm due to the formation of the Chl radical cation P680^+^ in (*a*) RC47-His and (*b*) non-oxygen-evolving PSII-His complexes were monitored. The decay kinetics were fitted to a sum of exponential decay components minimizing the sum of squares of the weighted residuals.

Although oxidation of tyrosine, Yz, has been demonstrated in the RC47 complex [22], the kinetics of electron transfer on the donor side of the complex have not been examined. The rate of reduction of P680^+^ was followed by measuring the change in absorbance at 830 nm following flash excitation (figure 5). In the case of oxygen-evolving PSII-His, the rate of P680^+^ reduction occurred with multi-exponential kinetics (data not shown) with the fastest phase occurring on the nano-second timescale, in line with results in the literature [35]. Both the RC47-His and the non-oxygen-evolving PSII-His complexes displayed similar but much slower rates of P680^+^ reduction, on the microsecond time-scale, with time constants as previously reported for non-oxygen-evolving PSII [36].

The 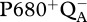
–P680Q_A_ difference spectra of RC47-His and PSII-His complexes were also recorded at 77 K (data not shown). They did not show significant differences, indicating the intactness of the reaction centre in the RC47-His complex. When normalized on an equal RC basis, assuming 35 Chl/PSII-His and 22 Chl/RC47-His, the amount of P680^+^ formed following flash excitation was within 15 per cent of each other, indicative of similar charge separation activity. As expected from previous studies, there was no evidence for retention of a functional Mn_4_CaO_5_ cluster or the secondary quinone electron acceptor, Q_B_, in the RC47-His complex [22].

**Figure 6.**
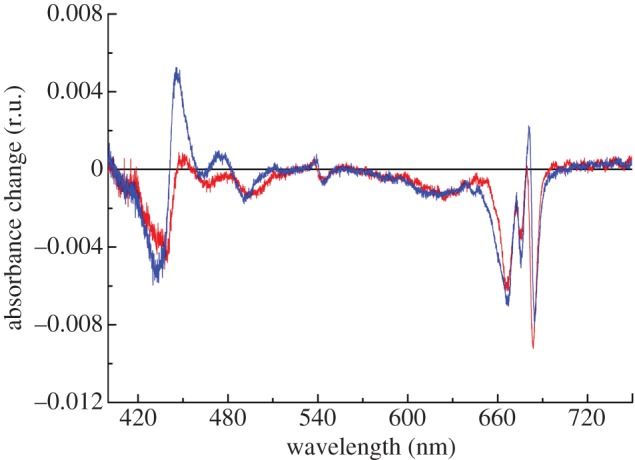
Light-minus-dark absorption spectrum at 78K induced by illumination of RC47-His and non-oxygen-evolving PSII-His complexes. The purified protein complexes were diluted to an OD of approximately 1 at the maximum in the Q_Y_-region and illuminated for approximately 1 min. Red, RC47-His; blue, PSII-His.

Light-minus-dark difference spectra were determined in RC47-His and PSII-His at 77 K to analyse the oxidation of secondary, side-path electron donors (cyt *b*-559, chlorophyll Z and β-carotene) that function in PSII at a low temperature (figure 6). The difference spectra were obtained by subtracting the absorbance spectrum in the dark-adapted state from that after illumination with continuous white light for about 60 s. The difference spectra exhibit the characteristic band shift centred at 543 nm (C550) owing to the reduction of Q_A_. An absorbance decrease at 557 nm due to light-induced oxidation of cyt *b*-559 at low temperature was not observed. Most likely cyt *b*-559 is already in the oxidized state before freezing. The remaining absorbance changes are consistent with the oxidation of chlorophyll Z (Chl_Z_) and/or β-carotene (Car). The observed band shift in the Q_Y_-region can be assigned to a blue shift of the site energy of the accessory Chl coordinated by D1 (Chl_D1_) caused by Car^+^ and 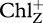 on the D2 side [37]. The additional bleaching band around 668 nm has been attributed to the oxidation of Chl_Z_ [37]. The features in the wavelength region between 460 and 510 nm indicate most likely the oxidation of Car that is oxidized directly by P680^+^. Subsequently, Car^+^ oxidizes cyt *b*-559 or Chl_Z_ if the cytochrome is preoxidized [38].

## 4. Discussion

We describe here further characterization of the RC47 assembly complex found in a *Δ*CP43 mutant blocked at a late stage in PSII assembly. Early work showed that the *Δ*CP43 mutant is unable to evolve oxygen in cells [21,22]. With hindsight, this effect is most likely due to the important role that CP43 plays in providing one of the amino-acid ligands to the Mn_4_CaO_5_ cluster [2] and the general destabilization of the cluster and the lumenal side of the complex that results in the absence of CP43. In addition, loss of CP43 also seems to impair electron transfer on the acceptor side of the PSII complex in cells [22], possibly by perturbing the conformation of the Q_B_-binding pocket [39].

Although incapable of oxidizing water, the isolated RC47 complex is able to photoreduce Q_A_ and photo-oxidize tyrosine Yz [22]. The kinetic experiments reported here on a His-tagged RC47 complex indicate that the rate of oxidation of Yz is equivalent to that seen in non-oxygen-evolving core complexes lacking the Mn_4_CaO_5_ cluster (figure 5), which suggests that the additional removal of CP43 has little impact on the local structure of PSII around P680^+^ and Yz. Consistent with this, previous measurements of the rate of charge recombination in isolated complexes suggested that loss of CP43 had only minor effects on the free energy difference between the 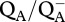 and 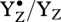 redox couples [22].

Many early attempts to isolate the RC47 complex for spectroscopic studies have relied on the stripping of CP43 from larger PSII core complexes, using chaotropic salts or detergents. However, the harsh extraction conditions used sometimes led to loss of Q_A_ [40–45] as well as removal of some pigment and LMM subunits [45]. Isolating the RC47 complex from mutants lacking CP43 helps overcome these potential problems [22].

By isolating the RC47-His assembly complex, we have been able to examine by mass spectrometry which of the LMM subunits of PSII are able to bind to the RC47 complex in the absence of CP43. As anticipated, the PsbZ, PsbK and Psb30 subunits, which bind to CP43 in the crystal structures and which are found in CP43 assembly sub-complexes [9,10], were not detected in RC47-His. The PsbJ subunit, which is located close to PsbK and Psb30 in the holoenzyme but not yet found in the CP43 sub-complex, was also not detected in RC47. These data are consistent with weaker binding of PsbJ to PSII in the absence of the CP43 sub-complex. As expected, all the LMM subunits intimately associated with the D1, D2 and CP47 subunits in the crystal structures (i.e. PsbI, PsbX, PsbE, PsbF, PsbY and PsbH) were identified. Interestingly all three LMM subunits—PsbT, PsbM and PsbL—that lie at the interface between the two monomeric complexes in the dimer were also present, despite the fact that the RC47-His was predominantly found in a monomeric form. These three subunits are considered important but not crucial for formation and stabilization of the dimeric form of the complex [46]. Presumably, additional monomer–monomer interactions, possibly involving lipid molecules [47] and the luminal extrinsic proteins [48], contribute strongly to stabilization of the dimer.

Our results therefore suggest that during disassembly of damaged PSII to form the damaged RC47 complex, CP43 might be detached in the form of a CP43 sub-complex consisting of CP43 and the LMM subunits PsbK, PsbZ and Psb30 [9,10]. The Psb27 subunit might also bind to the lumenally exposed region of disassembled CP43 [10]. All the remaining LMM subunits, apart from possibly PsbJ, may remain tightly bound to the damaged RC47 complex stabilizing bound pigment in those subunits that are undamaged and to be recycled.

The isolated RC47-His complex also contained the Psb28 accessory factor in agreement with earlier conclusions [26]. A binding site for Psb28 on the cytoplasmic side of CP47 close to PsbH has been suggested based on the ability of His-tagged Psb28 to pull-down unassembled CP47 and the reduction in Psb28 levels in a mutant lacking PsbH [26]. Although Psb28 is mainly associated with the RC47 complex in *Synechocystis* 6803, Psb28 can interact with larger monomeric WT complexes [26] and has recently been found in isolated core complexes containing Psb27 but lacking a fully functional Mn_4_CaO_5_ cluster [49,50]. The role of Psb28 is currently unknown, but on the basis of phenotype of a null mutant of *Synechocystis* 6803, it might regulate chlorophyll availability during the biogenesis of PSI and PSII [26]. We show here that a second Psb28 homologue, Psb28-2, also associates with RC47 (figure 2). Our current model is that Psb28 and Psb28-2 compete for the same binding site on RC47 and consequently do not bind to the same monomeric RC47 complex, assuming only one binding site. The reason for two Psb28 homologues in *Synechocystis* is unclear especially when many cyanobacteria have just one.

We have also detected the presence of ScpC and/or ScpD in the RC47 complex in accord with previous studies on high-light-treated WT cells [28,32]. Recent work has shown that binding of Scps to PSII is important for preventing the production of singlet oxygen most probably generated by chlorophyll molecules that are accidentally released either during PSII repair or during extensive PSII damage [51]. By taking into account the extremely fast turnover of the D1 protein in the *Δ*CP43 strain (figure 1 and [19]), we assume that even at standard growth irradiances, the probability of chlorophyll release is high and therefore the Scps are associated with RC47 even under these conditions.

We noticed that accumulation of the RC47 complex in cells of *Δ*CP43 was strongly dependent on the light conditions during growth. Upon lowering the irradiance from 40 to 5 µE m^−2^ s^−1^, the level of the RC47 complex dropped significantly (see the electronic supplementary material, figure S1). A similar decrease was reported by Shimada *et al.* [52] for *Δ*CP43 grown under light-activated heterotrophic growth conditions (growth in darkness with daily 15 min light exposure). Cellular chlorophyll levels were moderately reduced in *Δ*CP43 to approximately 70 per cent of WT levels, but this effect was observed at both light conditions (see the electronic supplementary material, figure S2). On the other hand, there was a strong decrease in phycobilin content in low-light-grown cells of the mutant in comparison with cells grown under standard conditions (see the electronic supplementary material, figure S2). This behaviour is reminiscent of the *Δ*PsbO mutant, which, like the *Δ*CP43 mutant, also exhibits an extremely fast turnover of the D1 protein [16,53].

We speculate that D1 turnover in these mutants might not be tightly coupled to light damage so that undamaged D1 is targeted for degradation at low light. In the case of RC47, the lack of CP43 might destabilize the N-terminal region of D1 and increase accessibility of the FtsH protease complex, which is involved is degrading photodamaged D1 during PSII repair [12], to the undamaged but destabilized D1 subunit. The lower levels of RC47 observed at low irradiance would reflect the inability of D1 synthesis and the light-driven biosynthesis of Chl to match this enhanced rate of D1 degradation [54]. Loss of chlorophyll induced by aberrantly high D1 turnover at low irradiance may lead to phycobilin deficiency [53,54]. For the PsbO-less mutant, fast turnover of D1 might be triggered at low light by spontaneous disassembly of the Mn_4_CaO_5_ cluster [53].

Because the RC47 complex is incapable of oxidizing water but is able to perform charge separation, it is potentially highly vulnerable to both donor-side and acceptor-side photoinhibition. Our analysis of the isolated RC47-His complex indicates that the vast majority of the complexes are photochemically active consistent with the operation of effective photoprotection/repair processes. The photoprotective mechanisms might include changes to the redox potential of Q_A_ so that singlet oxygen production following charge recombination is reduced [55], which to some extent might explain the observed impaired electron transfer on the acceptor side of the complex, between Q_A_ and Q_B_, in whole cells of *Δ*CP43 [22]; the operation of side-path electron donors Chl_Z_ and cyt *b*-559 in PSII [38] (figure 6); and impaired transfer of excitation energy from the phycobilisome to help ‘switch off’ the RC47 complex [52]. In addition, D1 is rapidly and preferentially labelled in the RC47 complex (figure 1; [19]) consistent with the view that RC47 complexes are excellent substrates for selective D1 replacement. Together, these features help ensure that only active RC47 complexes are available for assembling oxygen-evolving PSII.
